# Isoliquiritigenin in combination with visceral adipose tissue and related markers as a predictive tool for nonalcoholic fatty liver disease

**DOI:** 10.1007/s13105-023-00998-6

**Published:** 2023-11-24

**Authors:** Paola Mogna-Peláez, Ana Romo-Hualde, José I. Riezu-Boj, Fermin I. Milagro, David Muñoz-Prieto, José I. Herrero, Mariana Elorz, Alberto Benito-Boillos, J. Ignacio Monreal, Josep A. Tur, Alfredo Martínez, Itziar Abete, M. Angeles Zulet

**Affiliations:** 1 Department of Nutrition, Food Sciences and Physiology and Centre for Nutrition Research, Faculty of Pharmacy and Nutrition, Centre for Nutrition Research, University of Navarra, 31008 Pamplona, Spain; 2 Navarra Institute for Health Research (IdiSNA), 31008 Pamplona, Spain; 3 Biomedical Research Centre Network in Physiopathology of Obesity and Nutrition (CIBERobn), Instituto de Salud Carlos III, 28029 Madrid, Spain; 4 Liver Unit, Clínica Universidad de Navarra, 31008 Pamplona, Spain; 5 Biomedical Research Centre Network in Hepatic and Digestive Diseases (CIBERehd), 28029 Madrid, Spain; 6 Department of Radiology, Clínica Universidad de Navarra, 31008 Pamplona, Spain; 7 Clinical Chemistry Department, Clínica Universidad de Navarra, 31008 Pamplona, Spain; 8 Research Group on Community Nutrition and Oxidative Stress, University of Balearic Islands, 07122 Palma, Spain

**Keywords:** Isoliquiritigenin, Nonalcoholic fatty liver disease, Metabolomics, Insulin resistance, Visceral adipose tissue

## Abstract

Nonalcoholic fatty liver disease (NAFLD) is the most prevalent form of chronic liver disease in the world. New non-invasive diagnostic tools are needed to promptly treat this disease and avoid its complications. This study aimed to find key metabolites and related variables that could be used to predict and diagnose NAFLD. Ninety-eight subjects with NAFLD and 45 controls from the Fatty Liver in Obesity (FLiO) Study (NCT03183193) were analyzed. NAFLD was diagnosed and graded by ultrasound and classified into two groups: 0 (controls) and ≥ 1 (NAFLD). Hepatic status was additionally assessed through magnetic resonance imaging (MRI), elastography, and determination of transaminases. Anthropometry, body composition (DXA), biochemical parameters, and lifestyle factors were evaluated as well. Non-targeted metabolomics of serum was performed with high-performance liquid chromatography coupled to time-of-flight mass spectrometry (HPLC-TOF-MS). Isoliquiritigenin (ISO) had the strongest association with NAFLD out of the determinant metabolites. Individuals with higher concentrations of ISO had healthier metabolic and hepatic status and were less likely to have NAFLD (OR 0.13). Receiver operating characteristic (ROC) curves demonstrated the predictive power of ISO in panel combination with other NAFLD and IR-related variables, such as visceral adipose tissue (VAT) (AUROC 0.972), adiponectin (AUROC 0.917), plasmatic glucose (AUROC 0.817), and CK18-M30 (AUROC 0.810). Individuals with lower levels of ISO have from 71 to 82% more risk of presenting NAFLD compared to individuals with higher levels. Metabolites such as ISO, in combination with visceral adipose tissue, IR, and related markers, constitute a potential non-invasive tool to predict and diagnose NAFLD.

**Supplementary Information:**

The online version contains supplementary material available at 10.1007/s13105-023-00998-6.

## Introduction

Nonalcoholic fatty liver disease (NAFLD) is defined as the presence of steatosis in at least 5% of hepatocytes in the absence of excessive alcohol intake [8]. Currently, NAFLD represents the most common form of chronic liver disease in the world, with a continuously growing prevalence of approximately 30% of the overall population [35, 62].

NAFLD is greatly associated with obesity and its related comorbidities, such as type 2 diabetes mellitus, insulin resistance, dyslipidemias, and cardiovascular disease. Forsooth, NAFLD may be considered the hepatic manifestation of metabolic syndrome [79]. NAFLD encompasses simple steatosis and non-alcoholic steatohepatitis (NASH), its inflammatory phenotype. The progress of the disease to NASH is strongly related to an increased risk of fibrosis, cirrhosis, end-stage liver disease, and hepatocellular carcinoma [62].

NAFLD’s pathogenesis is complicated and multifactorial, involving individual elements such as lifestyle factors, genetics, and gut microbiota composition [13]. Nevertheless, increased inflammation and oxidative stress are among the main physio-pathological mechanisms involved in the development and progression of the disease [64, 76].

Liver biopsy is considered the gold-standard diagnostic methodology for NAFLD; however, this technique is invasive and expensive and cannot be applied as a routine checkup in the population [30]. Subrogate routine markers (transaminases), several scores and indexes (fatty liver index, FLI), and imaging techniques, such as magnetic resonance imaging (MRI) and ultrasonography, have also been proposed to diagnose this disease [7]. Nevertheless, NAFLD continues to be a silent epidemic that is manifested when there are advanced stages of fibrosis [59]. In this regard, there is increasing interest in finding non-invasive diagnostic markers that could help in the early diagnosis of this disease [35].

An early diagnosis of NAFLD with non-invasive methods may be a key factor in treating promptly this disease and avoiding its many complications [34]. In this sense, omics technologies represent an excellent tool, particularly helpful for the early diagnosis of several diseases [48, 63]. Recently, metabolomics has gained attention in the field of metabolic diseases, not only because of its ability to elucidate biomarkers but because, since metabolites directly influence metabolism, they could contribute to explain the pathophysiology of such diseases as well [27–29, 74].

With this background, this study aimed to identify and deepen the knowledge about metabolites and other non-invasive markers that could be used to predict NAFLD. This was achieved using omics technologies and more specifically non-targeted metabolomics.

## Materials and methods

### Study participants

The current research includes the assessment of baseline measurements of 98 participants with NAFLD, recruited for the FLiO (Fatty Liver in Obesity) study (NCT03183193), a randomized controlled trial that included adults between the ages of 40 and 80 years old, who were overweight or obese (BMI between 27.5 and 40 Kg/m^2^) and presented NAFLD diagnosed by ultrasonography. The control group consists of 45 individuals who were recruited as a part of the EHGNA study (a continuation of FLiO), presented similar age range and sex distribution as the NAFLD subjects but, contrarily to the NAFLD group, presented normal weight (BMI < 25 Kg/m^2^) and did not have fatty liver. Exclusion criteria included elevated alcohol consumption (> 21 units of alcohol per week for men and > 14 units for women), presence of known liver disease, endocrine disorders (hyperthyroidism or uncontrolled hypothyroidism), weight loss > 3 Kg in the past 3 months, the use of weight modifiers, severe psychiatric disorders, active autoimmune diseases, acute infections, pharmacological treatment with drugs that could cause hepatic steatosis or alteration in hepatic tests (immunosuppressants, cytotoxic agents, corticosteroids), the inability to follow the diet (food allergies/intolerances) and difficulty in following the scheduled visits. The study protocol was approved by the Research Ethics Committee of the University of Navarra (ref. 54/2015). All the procedures were done in accordance with the declaration of Helsinki. All the participants gave written informed consent prior to their inclusion in the study.

### Anthropometric, body composition, and biochemical evaluation

Anthropometric measurements, body composition, and blood pressure were determined under fasting conditions in the Metabolic Unit of the University of Navarra, as previously described [11]. Body mass index (BMI) was calculated as the body weight of the subject measured in Kilograms divided by the squared height measured in meters (Kg/m^2^). Body composition, including visceral adipose tissue (VAT), was determined by dual-energy X-ray absorptiometry (DXA) following the instructions of the manufacturer (Lunar iDXA, enCORE 14.5, Madison, WI).

Blood samples were collected under 8–10 h fasting conditions. The samples were processed to obtain biochemical determinations in the biochemistry laboratory of the University of Navarra Clinic (CUN). Blood triglycerides (TG), total cholesterol, high-density lipoprotein cholesterol (HDL-c), glucose, and insulin were quantified on an autoanalyzer with specific commercial kits and following the instructions of the company (Cobas 8000, Roche Diagnostics, Switzerland). Low-density lipoprotein cholesterol (LDL-c) was calculated using the Friedewald formula [17].

The Homeostatic model assessment of insulin resistance (HOMA-IR) was calculated as $\frac{\textrm{fasting}\ \textrm{insulin}\ \left(\frac{\upmu \textrm{U}}{\textrm{mL}}\right)\times \textrm{fasting}\ \textrm{glucose}\ \left(\textrm{mmol}/\textrm{L}\right)\ }{22.5}$ [58]. The Castelli’s risk index (CRI) was calculated as $\frac{TC}{HDL-c}$, while the atherogenic index of plasma (AIP) was calculated as $\log \frac{TG}{HDL-c}$ , as previously described [16, 39]

Retinol binding protein (RBP4), leptin, and adiponectin were analyzed and quantified using specific ELISA kits (Demeditec; Kiel-Wellsee, Germany) in a Triturus autoanalyzer (Grifols, Barcelona, Spain) at the biochemistry laboratory of the Center for the Nutrition Research at the University of Navarra (CIN, Pamplona, Spain). Leukocyte cell-derived chemotaxin-2 (LECT2) was analyzed and quantified using the same Triturus autoanalyzer with specific kits for this chemotaxin (Biovendor LLC, NC, USA).

### Assessment of liver status

The entire hepatic assessment was performed by qualified professionals of the CUN under fasting conditions. The diagnosis of NAFLD was determined by ultrasonography (Siemens ACUSON S2000 and S3000), as described elsewhere [44]. The participants were classified into two groups: 0 (controls) and ≥ 1 (NAFLD). MRI (Siemens Aera 1.5T) determined liver fat content by the Dixon technique [6]. Aspartate aminotransferase (AST), alanine aminotransferase (ALT), and gamma-glutamyl transferase (GGT) were quantified on an autoanalyzer with specific commercial kits and following the instructions of the company (Cobas 8000, Roche Diagnostics, Switzerland). M30 and M65, which are considered hepatic fibrosis markers [36], were quantified with an enzyme-linked immunosorbent assay (ELISA) method using commercial kits (PEVIVA, Bromma, Sweden) according to the manufacturer’s instructions. The fatty liver index (FLI) was computed using a previously described formula [3], that considers serum triglycerides, BMI, waist circumference, and GGT concentrations to determine the probability of suffering NAFLD.

### Metabolomics

Non-targeted serum metabolomics were carried out in the Metabolomics Unit of the University of Navarra. Sample pre-processing consisted in defrosting and homogenizing the samples that were stored at −80 °C. A total of 150 μL aliquots of serum were prepared, and 450 μL of methanol (MeOH, grade LC–MS, Scharlab, Sentemant, Spain) was added to each aliquot. Then, samples were vigorously vortexed for 2 min (VX-2500 multi-tube vortexer, VWR, PA, USA) and centrifuged for 10 min at 10000 rmp (Biofuge A, Heraeus Sepatech, Germany). The supernatant was collected, evaporated under nitrogen flux (Turvovap® LV, Caliper LifeSciences, Waltham, MA, USA), and recovered in 150 μL of H_2_O: MeOH (5:95 V:V). Then, the samples were analyzed under High performance liquid chromatography (HLPC; Agilent Technologies 1200) equipped with a Time of flight (TOF) mass detector (Agilent Mass Accuracy 6220) (LC–MS), operated in positive electrospray ionization mode (ESI+) and negative mode (ESI−). The stationary phase used was a Zorbax SB-C18 column (Agilent Technologies), and the mobile phase consisted of 0.1% formic acid in water (A) and 0.1% formic acid in MeOH (B). The gradient elution was 0% B, 0–5min; 0–100% B, 5–20 min; 100% B, 20–25 min; 100–0% B, 25–30 min. After the analyses, the column was re-equilibrated for 2 min at = % B. The injection volume was 20 μL and the flow rate was 0.5 mL min^−1^. Chromatography was performed at 40 °C. The ESI conditions were as follows: gas temperature, 350 °C; drying gas, 10 L min^−1^; nebulizer, 45 psig; capillary voltage, 3500 V; fragmentor, 17 V; and skimmer, 65 V. The instrument was set out to acquire over the m/z range 100–2000 with an acquisition rate of 1.03 spectra per s. To evaluate the quality in this analysis, three types of quality control samples (QCs) were used: (i) column test, (ii) pool plasma prepared by mixing equal volumes from each of the samples, (iii) pool spiked plasma prepared by mixing pool plasma with L-alanine, glycine, L-phenylalanine, citric acid, L-glutamic acid, caffeine, and leucine (Sigma-Aldrich, MA, USA). The analytical procedure has been previously described [2, 10].

Chromatograms were processed with MassHunter Qualitative Analysis B.06.00 software (Agilent Technologies, CA, USA) to ensure quality. Metabolite alignment was obtained with the XCMS Online software (The Scripps Research Institute, La Jolla, CA 92037, USA) attending to the mass-to-charge ratio and the retention time. The alignment used a 0.2 min-retention time and a 5 mDalton mass tolerance window. The peak intensity of the metabolites was controlled by a logarithmic transformation and monitored by Pareto scaling. Partial least squares discriminant analyses (PLSDA), Random forest, Volcano plots, and other multivariate tools were conducted in the MetaboAnalyst Software version 5.0 (Xia Lab of McGill University, Quebec, Canada) to obtain key metabolites differentiating the control and NAFLD groups. PLSDA determined the Variable of value of variable importance in projection (VIP). Metabolites with a VIP score value greater than 1.0 were chosen for proof of identity. Finally, the metabolite identification was carried out using the Metlin database (The Scripps Research Institute). The adducts used for the search criteria in the positive polarity were [M+H]^+^, [M+Na]^+^, and [M+H-H_2_O]^+^, while for the negative polarity were [M-H]^−^ and [M-H-H_2_O]^−^, both considering a 5 mDalton mass tolerance window. Metabolites were expressed and analyzed according to their intensity measured in arbitrary units (AU), which carries a direct relation with its concentration. Identification of ISO (Sigma-Aldrich, MA, USA) was confirmed by analyzing commercial standards in identical conditions of samples.

### Lifestyle assessment (dietary intake and physical activity)

Information about dietary intake and physical activity was extracted from validated questionnaires. Diet was assessed with a semiquantitative food frequency questionnaire (FFQ) of 137 items, previously validated in Spain for energy and nutrient intake [15]. The nutrient composition of the food items was derived from accepted Spanish food composition tables [18, 61]. Mediterranean diet adherence was evaluated using a 17-point screening questionnaire, whose final score ranged between 0 and 17, with a higher punctuation indicating better adherence [19, 46].

### Statistical analyses

All statistical analyses were performed using Stata software version 12.0 (StataCorp, College Station, TX, USA). The normal distribution of each variable was evaluated using the Shapiro–Wilk tests. Parametrical tests were used for variables with a normal distribution, while non-parametrical tests were used for those which not. *T*-tests and Wilcoxon tests were used for numerical variables, and chi-squared tests were used for categorical variables to compare baseline characteristics of controls and subjects with NAFLD. Participants were then classified according to ISO intensity tertiles (T1: 8247.65-63573.35; T2: 63658.64-120638.35; and T3: 121582.47-1544021.9). Body composition, hepatic status, and insulin resistance markers among the tertiles were evaluated by Kruskal–Wallis tests for numerical variables and chi-squared tests for categorical variables. Spearman correlations were performed to evaluate the association between hepatic status, metabolites, insulin resistance, and cardiovascular risk markers. Bootstrap stepwise multivariable regressions were performed to select the metabolites that were further associated with the disease. Univariate and multivariate logistic regressions were performed with the presence of NAFLD as the dependent variable. Receiver operating characteristic curve analyses (ROC) and the areas under the ROC curve (AUROC) were calculated to evaluate the power of prediction and the diagnostic performance of several variables for liver fat. Validation of these results was performed by calculating the optimism-corrected value using Tibshirani’s enhanced bootstrap method described by Harrell [23]. All *p*-values presented are two-tailed. Statistical significance was considered at *p* < 0.05. Relative risk (RR) was calculated from OR using a previously described formula [47]

## Results

### General baseline characteristics

Body composition, hepatic status, insulin resistance, cardiovascular risk, and inflammatory markers of the study participants are presented in Table 1 (NAFLD group, *n* = 98; control group, *n* = 45). In the NAFLD group, 43 subjects were women and 55 were men. In the control group, 29 subjects were women and 16 were men (*p* = 0.022). The mean age of the control group was 50.34 (SD = 9.24) years, while in the NAFLD group was 48.77 (SD = 5.50); no significant differences were found regarding the age of the participants (*p* = 0.302).

**Table 1:** Baseline characteristics, body composition, hepatic status, insulin resistance, cardiovascular risk, and inflammatory markers of the Control and NAFLD groups of the study

	Control group (*n* = 45)	NAFLD group (*n* = 98)	*p*-value
Body composition			
BMI (Kg/m^2^)	23.3 (2.5)	33.5 (3.7)	< 0.001
Body fat (%)	30.99 (9.05)	42.47 (6.07)	< 0.001
Waist circumference (cm)	78.78 (9.10)	109.03 (9.36)	< 0.001
VAT (Kg)	0.5 (0.4)	2.3 (1.0)	< 0.001
Hepatic status			
ALT (IU/L)	21.49 (14.34)	33.20 (17.25)	< 0.001
AST (IU/L)	23.63 (10.92)	24.67 (9.65)	0.212
GGT (IU/L)	27.26 (38.48)	37.19 (26.63)	< 0.001
Steatosis degree	0 (0)	1.47 (0.64)	< 0.001
Liver fat (%)	3.62 (1.93)	7.22 (5.29)	< 0.001
FLI index	17.00 (18.10)	78.81 (18.65)	< 0.001
M30 (U/L)	40.77 (23.36)	94.49 (72.32)	< 0.001
M65 (U/L)	114.70 (67.09)	147.76 (97.48)	0.046
Insulin resistance and cardiovascular risk, and inflammatory markers			
Glucose (mg/dL)	90.73 (6.49)	102.57 (15.76)	< 0.001
Insulin (mg/dL)	4.16 (1.79)	17.01 (8.43)	< 0.001
HOMA-IR	0.94 (0.43)	4.41 (2.45)	< 0.001
Total cholesterol (mg/dL)	218.00 (5.99)	194.34 (37.49)	< 0.001
LDL cholesterol (mg/dL)	133.51 (4.81)	116.11 (3.44)	0.004
HDL cholesterol (mg/dL)	67.76 (17.65)	52.89 (13.18)	< 0.001
Atherogenic Index CRI	3.375 (0.134)	3.876 (0.121)	0.013
Atherogenic Index AIP	0.070 (0.033)	0.354 (0.026)	< 0.001
Triglycerides (mg/dL)	83.57 (38.16)	128.83 (63.45)	< 0.001
Leptin (ng/ml)	14.14 (11.52)	37.96 (29.05)	< 0.001
Adiponectin (ug/ml)	14.02 (5.72)	6.65 (2.19)	< 0.001
LECT2 (ng/ml)	23.83 (9.04)	41.57 (10.10)	< 0.001
RBP4 (mg/l)	26.69 (7.03)	35.34 (9.87)	< 0.001
Lifestyle assessment (dietary intake and physical activity)			
Physical activity	1.0 (1.0)	1.3 (1.00)	0.202
MedDiet adherence score	9.9 (2.5)	5.9 (1.9)	< 0.001
Total energy (Kcal/day)	2232 (583)	2602 (952)	0.026
Carbohydrates (TEV%)	36.9 (8.2)	43.0 (6.9)	< 0.001
Proteins (TEV%)	18.7 (3.5)	17.2 (3.9)	0.005
Lipids (TEV%)	42.5 (8.3)	37.0 (7.0)	< 0.001
MUFA (TEV%)	21.2 (5.9)	17.6 (4.5)	< 0.001
PUFA (TEV%)	7.7 (2.4)	5.5 (1.7)	< 0.001
SFA (TEV%)	11.9 (2.3)	10.6 (2.5)	0.063
Fiber (g/day)	24.9 (10.1)	23.8 (8.6)	0.637
Glycemic load	111.3 (41.7)	155.4 (44.0)	< 0.001
Sodium (mg/day)	2046 (665)	2692 (1015)	< 0.001

Values are expressed as mean (SD)

Abbreviations: *AIP*, atherogenic index of plasma; *ALT*, alanine aminotransferase; *AST*, aspartate aminotransferase; *BMI*, body mass index; *CRI*, Castelli’s risk index; *FLI*, fatty liver index; *GGT*, gamma-glutamyl transferase; *HDL*, high density lipoprotein; *HOMA-IR*, homeostatic model assessment for insulin resistance; *LDL*, low density lipoprotein; *MedDiet score*, Mediterranean diet adherence score; *MUFA*, monounsaturated fatty acids; *NAFLD*, nonalcoholic fatty liver disease; *PUFA*, polyunsaturated fatty acids; *RBP4*, retinol binding protein 4; *SFA*, saturated fatty acids; *TEV*, total energy value; *VAT*, visceral adipose tissue

Body composition measurements significantly differed between the groups, clearly suggesting that the NAFLD subjects present worst metabolic health and hepatic status. Glucose, insulin, HOMA-IR, triglycerides, LECT2, RBP4, and leptin were significantly increased in individuals with NAFLD. As expected, adiponectin was increased in controls. Total cholesterol and LDL cholesterol levels were significantly higher in the control group compared to the NALFD group (*p* < 0.001 and *p* = 0.004, respectively). However, both calculated atherogenic indexes, CRI and AIP, were significantly higher in the NAFLD group (*p* = 0.013 and *p* < 0.001). Moreover, several of these markers significantly correlated with hepatic fat percentage. Concretely, LECT2, RBP4, M30, and M65 were positively correlated with hepatic fat, while adiponectin was negatively correlated with hepatic fat (*p* < 0.001).

### Metabolomics

**Fig. 1:**
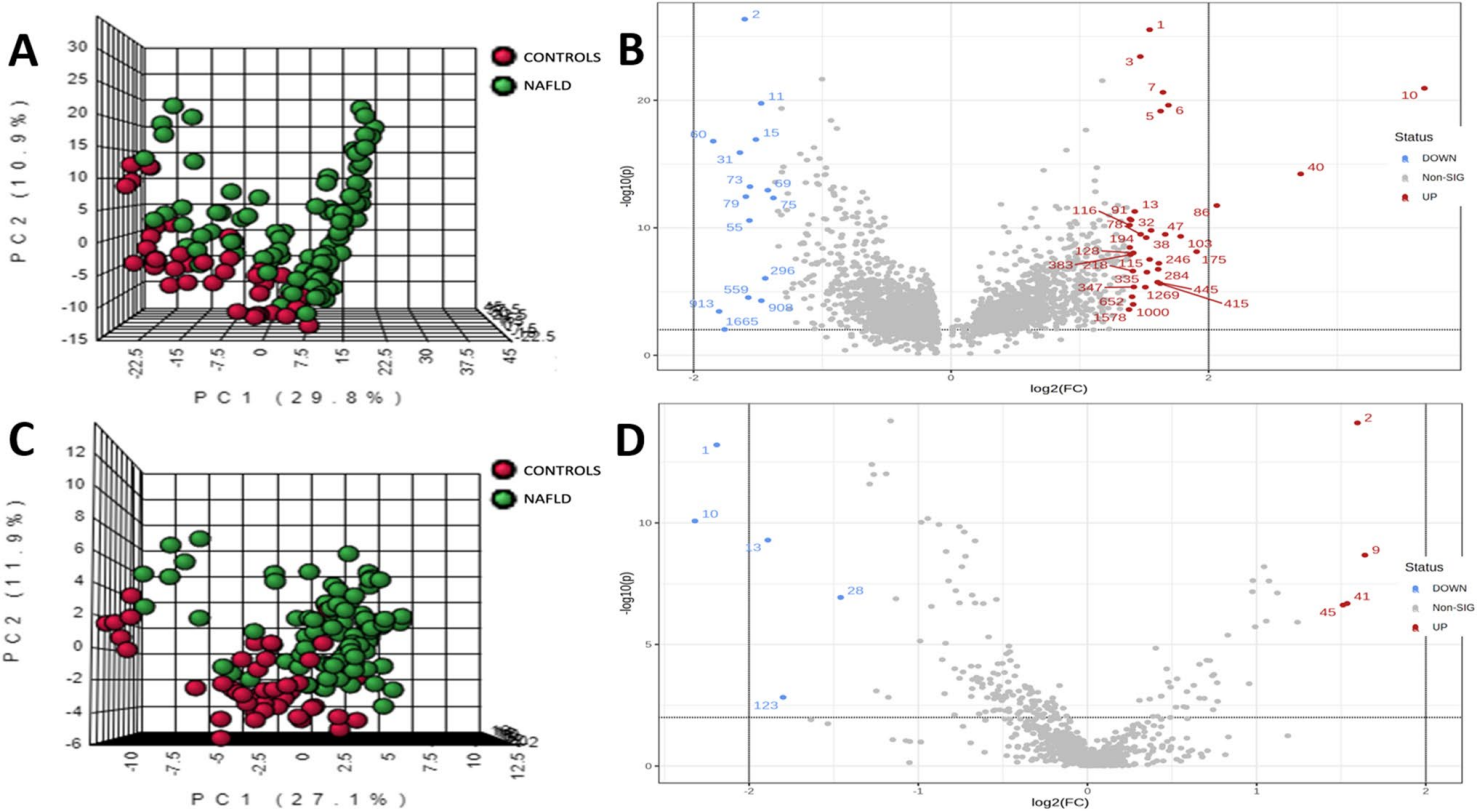
Metabolomic fingerprint of controls and NAFLD subjects. **A**. Principal component analysis (PCA) in positive polarity of controls and NAFLD subjects. **B**. Volcano plot in positive polarity of controls and NAFLD subjects. **C**. Principal component analysis (PCA) in negative polarity of controls and NAFLD subjects. **D**. Volcano plot in negative polarity of controls and NAFLD subjects

HPLC-TOF-MS method allowed the detection of 6599 features in the ESI+ mode and 1036 features in the ESI- mode (data not shown). The discriminant analysis determined a total of 57 discriminant metabolites between the NAFLD and control groups (Fig. 1), 48 with positive polarity and 9 with negative polarity (Supplementary table 1). After their identification using the Metlin database, the metabolites were subjected to bootstrap stepwise regressions to select those with the most predictive capacity for NAFLD. A total of four metabolites were selected: vignatic acid A, perseitol, 4-(2-Nitroethyl) phenyl primeveroside, and ISO. After adjusting for other important variables and possible confounders, ISO was selected as the primary determinant metabolite, because of its predictive ability towards NAFLD.

ISO negatively correlated with key anthropometric measurements as well, such as waist circumference (*p* < 0.001), BMI (*p* < 0.001), VAT (*p* < 0.001), and body fat (*p* < 0.001). Moreover, ISO was significantly correlated with biochemical parameters involved in NAFLD. Concretely, the metabolite was positively correlated with HDL cholesterol (*p* = 0.015), while it was negatively correlated with triglycerides (*p* = 0.026), insulin (*p* < 0.001), and HOMA-IR (*p* < 0.001).

ISO was significantly correlated with cardiovascular risk and adiposity markers as well. Specifically, the metabolite was positively correlated with adiponectin (*p* < 0.001); while it was negatively correlated with LECT2 (*p* = 0.002), RBP4 (*p* = 0.011), leptin (*p* = 0.002), and fibrosis markers M30 and M65 (*p* = 0.005 and *p* = 0.044, respectively).

The study population was divided into tertiles according to their ISO intensity, which is a direct representation of their concentration. The first tertile consists of those individuals with the lowest levels of the metabolite, while the third tertile encompasses the individuals with the highest levels of ISO (T1: 8247.65-63573.35; T2: 63658.64-120638.35; and T3: 121582.47-1544021.9). The sex distribution in the tertiles (male/female) were 27/21, 20/28, and 24/23, respectively, with no significant differences between them (*p* = 0.350).

Body composition, hepatic status, insulin resistance, and cardiovascular risk markers were evaluated and compared among the tertiles as well (Table 2). BMI, waist circumference, VAT, body fat percentage, insulin, HOMA-IR, LECT2, RBP4, and leptin were significantly decreased in the third tertile of the studied group. Similarly, ALT, steatosis degree, hepatic fat, FLI index, M30, and M65 markers were reduced as well in individuals with higher ISO intensity. Contrarily, adiponectin was increased in the third tertile. Curiously, total cholesterol and LDL cholesterol were increased in the third tertile of the population as well; nevertheless, the AIP atherogenic index was significantly decreased in this tertile.

**Table 2:** Baseline characteristics, body composition, hepatic status, insulin resistance, cardiovascular risk, and inflammatory markers according to Isoliquiritigenin intensity tertiles.

	T1 (*n* = 48)	T2 (*n* = 48)	T3 (*n* = 47)	*p*-value
Body composition				
BMI (Kg/m^2^)	32.25 (29.38–35.98)	31.55 (27.45–34.34)	26.88 (22.80–31.99)	< 0.001 #Ω
Body fat (%)	41.6 (36.3–46.8)	40.7 (35.8–46.7)	35.8 (30.0–41.3)	< 0.001 #
Waist circumference (cm)	106.5 (96.35–118.5)	105.2 (92.0–110.0)	94.5 (73.5–108.9)	< 0.001#Ω
VAT (Kg)	1.98 (1.09–3.06)	1.79 (1.00–2.34)	1.06 (0.19–2.22)	0.006#Ω
Hepatic status				
ALT (IU/L)	31.0 (22.0–46.5)	22.0 (16.9–31.4)	19.2 (14.5–35.0)	0.011*
AST (IU/L)	25.0 (19.0–29.0)	21.0 (18.0–26.9)	20.9 (17.0–27.7)	0.169
GGT (IU/L)	26 (17–43)	24 (18–38)	20 (15–38)	0.420
Steatosis degree	1 (1–2)	1 (0.5–2)	0 (0–1)	< 0.001#
Liver fat (%)	6.6 (3.3–12.4)	4.2 (3.0–7.2)	3.8 (2.3–5.4)	< 0.001*#
FLI index	82.71 (48.82–93.18)	75.92 (32.32–90.10)	47.73 (7.03–78.34)	< 0.001#Ω
M30 (U/L)	71.34 (47.39–150.38)	52.35 (34.21–96.99)	45.73 (30.32–81.80)	0.024*#
M65 (U/L)	134.77 (98–197.25)	117.36 (81.65–167.54)	102.85 (79.32–125.68)	0.045#
Insulin resistance, cardiovascular, and inflammatory risk markers				
Glucose (mg/dL)	97.3 (89.3–105.0)	96.1 (90.5–104.0)	94.0 (87.9–101.0)	0.331
Insulin (mg/dL)	13.75 (7.84–20.85)	11.75 (6.45–19.75)	5.60 (3.37–16.30)	< 0.001#Ω
HOMA-IR	3.39 (1.85–5.71)	3.13 (1.53–5.08)	1.40 (0.74–3.96)	< 0.001#Ω
Total cholesterol (mg/dL)	186 (153–209)	212 (184–234)	214 (181–238)	0.003#
LDL cholesterol (mg/dL)	105.0 (86.8–135.0)	129.8 (105.0–151.7)	134.4 (99.6–150.0)	0.005#
HDL cholesterol (mg/dL)	50.5 (44.5–61.0)	58.0 (45.9–65.9)	56.8 (47.0–77.0)	0.050
Atherogenic index CRI	3.46 (2.73–4.54)	3.62 (3.07–4.46)	3.28 (2.78–4.27)	0.494
Atherogenic index AIP	0.32 (0.09–0.52)	0.29 (0.04–0.46)	0.08 (−0.06–0.39)	0.026#Ω
Triglycerides (mg/dL)	117.5 (72.0–165.5)	112.5 (74.5–133.5)	88.0 (67.0–121.0)	0.083
Leptin (ng/ml)	28.1 (17.6–42.6)	25.6 (13.6–42.0)	16.4 (10.0–27.5)	0.003#Ω
Adiponectin (ug/ml)	6.22 (4.66–9.91)	7.17 (5.44–10.09)	8.82 (6.12–13.47)	0.005#Ω
LECT2 (ng/ml)	42.9 (31.9–49.4)	36.2 (27.4–42.4)	30 (23.2–43.8)	0.023#
RBP4 (mg/l)	33.8 (26.7–42.8)	30.9 (27.9–38.1)	29.0 (23.7–34.3)	0.018#Ω
Lifestyle assessment (dietary intake and physical activity)				
Physical Activity	1 (0–2)	1 (0–2)	1 (0–2)	0.545
MedDiet score	6 (4–7)	7 (5–8)	8 (6–11)	< 0.001*#
Total energy (Kcal/day)	2572 (2062–3193)	2457 (1845–2894)	2173 (1702–2647)	0.035#
Carbohydrates (TEV%)	40.7 (36.3–45.8)	44.0 (38.3–49.5)	40.4 (34.0–43.0)	0.012Ω
Proteins (TEV%)	15.9 (15.1–18.4)	17.8 (13.6–19.5)	17.6 (16.1–19.5)	0.030*#
Lipids (TEV%)	40.7 (35.3–44.6)	34.9 (31.8–41.4)	39.5 (33.8–43.8)	0.038*
MUFA (TEV%)	18.8 (16.3–21.2)	17.7 (14.4–20.0)	19.2 (15.3–22.7)	0.102
PUFA (TEV%)	5.6 (4.7–7.2)	5.4 (4.5–7.0)	6.5 (4.8–7.5)	0.344
SFA (TEV%)	11.2 (10.0–12.7)	9.9 (8.9–11.1)	10.7 (9.8–12.6)	0.016*Ω
Fiber (g/day)	22.3 (16.9–28.3)	24.9 (19.3–30.3)	22.4 (18.3–28.3)	0.356
Glycemic load	143.0 (111.3–176.6)	142.4 (96.9–185.2)	117.5 (68.5–148.5)	0.015Ω#
Sodium (mg/day)	2601 (1936–3286)	2386 (1843–2786)	2182 (1515–2675)	0.033#

Values are expressed as median (interquartile range)

Significant differences: #significant *p*-values between T1 vs T3, *significant *p*-value between T1 and T2, Ω Significant *p*-value between T2 and T3

*AIP*, atherogenic index of plasma; *ALT*, alanine aminotransferase; *AST*, aspartate aminotransferase; *BMI*, body mass index; *CRI*, Castelli’s risk index; *FLI*, fatty liver index; *GGT*, gamma-glutamyl transferase; *HDL*, high density lipoprotein; *HOMA-IR*, homeostatic model assessment for insulin resistance; *LDL*, low density lipoprotein; *MedDiet score*, Mediterranean diet adherence score; *MUFA*, monounsaturated fatty acids; *NAFLD*, nonalcoholic fatty liver disease; *PUFA*, polyunsaturated fatty acids; *RBP4*, retinol binding protein 4; *SFA*, saturated fatty acids; *TEV*, total energy value; *VAT*, visceral adipose tissue

### Logistic regressions

To further evaluate the predictive capacity and possible functionality of ISO as a predictor for NAFLD, logistic regressions with their respective odds ratios (OR) and ROC curves analyses were calculated. A univariate analysis between the presence of NAFLD and ISO tertiles demonstrated that the metabolite was significantly able to predict the disease (T2: OR = 0.60, 95% confidence interval = 0.22–1.63, *p* = 0.317; T3: OR = 0.17, 95% confidence interval = 0.06–0.45, *p* < 0.001) with a moderate capacity (AUROC 0.687, Table 3). Other contributing factors were evaluated and chosen based on bootstrap stepwise regressions as well, of which the ones with the most predictive capacity for the presence of NAFLD were VAT, adiponectin, plasmatic glucose, and M30. These results presented high statistical significance as well (*p* < 0.001).

**Table 3:** Logistic regressions between NAFLD as the dependent factor and Isoliquiritigenin tertiles as predictive factors

	Univariate ISO analysis			Model 1 ISO and Visceral Adipose Tissue			Model 2 ISO and adiponectin			Model 3 ISO and plasmatic glucose			Model 4 ISO and M30		
NAFLD	OR (95% CI)	*p*-value	AUROC	OR (95% CI)	*p*-value	AUROC	OR (95% CI)	*p*-value	AUROC	OR (95% CI)	*p*-value	AUROC	OR (95% CI)	*p*-value	AUROC
ISO T1 (reference) (*n* = 48)	1.00		0.687 (0.687†)	1.00		0.965 (0.964†)	1.00		0.910 (0.904†)	1.00		0.831 (0.823†)	1.00		0.821 (0.813†)
ISO T2 (*n* = 48)	0.60 (0.22–1.63)	0.315		0.39 (0.07–2.02)	0.265		0.72 (0.18–2.88)	0.647		0.50 (0.16–1.54)	0.229		0.68 (0.22–2.11)	0.513	
ISO T3 (*n* = 47)	0.17 (0.06–0.45)	0.001		0.16 (0.02–0.89)	0.037		0.20 (0.05–0.75)	0.017		0.14 (0.04–0.44)	0.001		0.19 (0.06–0.59)	0.004	
Adjusted models (sex, age, and physical activity)															
ISO T1 (reference) (*n* = 48)	1.00		0.750 (0.735†)	1.00		0.979 (0.972†)	1.00		0.931 (0.917†)	1.00		0.841 (0.817†)	1.00		0.835 (0.810†)
ISO T2 (*n* = 48)	0.58 (0.20–1.67)	0.315		0.19 (0.02–1.68)	0.137		0.47 (0.10–2.16)	0.340		0.54 (0.16–1.75)	0.309		0.71 (0.21–2.37)	0.581	
ISO T3 (*n* = 47)	0.13 (0.04–0.37)	0.001		0.17 (0.01–2.12)	0.170		0.10 (0.02–0.47)	0.003		0.15 (0.04–0.47)	0.001		0.17 (0.05–0.57)	0.004	

*AUROC*, area under the receiver operating characteristic curve; *NAFLD*, nonalcoholic fatty liver disease; *OR*, odds ratio; *95% CI*, 95 percent confidence interval

*p*-value for the isoliquiritigenin in the logistic regression model. Adjusted models are adjusted by sex, age, and physical activity

†Optimism-corrected AUROC value

**Fig. 2:**
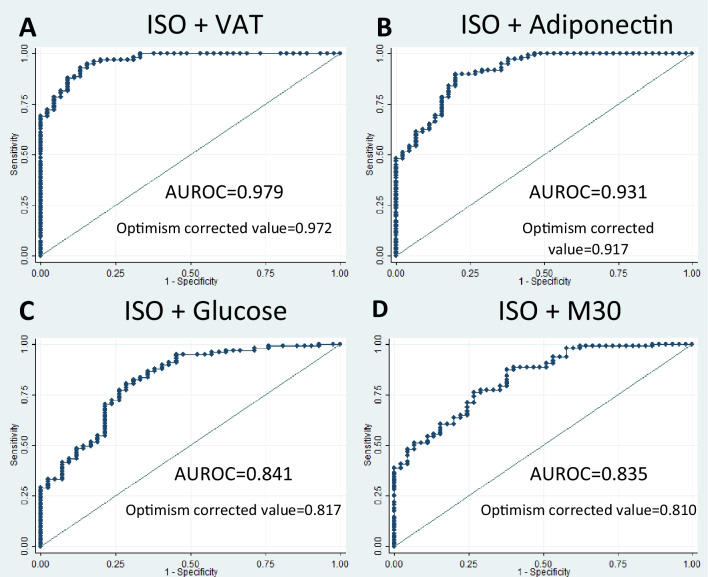
Receiver operating characteristic curves for nonalcoholic fatty liver disease. **A** Isoliquiritigenin and visceral adipose tissue. **B** Isoliquiritigenin and adiponectin. **C** Isoliquiritigenin and M30. **D** Isoliquiritigenin and plasmatic glucose

After selecting these contributing variables, multivariable logistic regressions and ROC curve analyses were performed to evaluate ISO alongside a panel of other variables (Table 3). The regressions were then adjusted with sex, age, and physical activity. Age and physical activity did not seem to explain or predict NAFLD in any of the models. Sex did seem to have a significant effect, but only on the prediction of NAFLD in the VAT and ISO model. Model 1 combined ISO with VAT. Model 2 combined ISO with adiponectin. Model 3 combined ISO with plasmatic glucose, and Model 4 combined ISO with M30. ISO continued to have a significant predictive value even in combination with these potent contributing variables. The AUROC results were validated using the optimism-corrected value previously mentioned and are shown in Table 3 (Fig. 2). Additionally, RR was estimated from the OR obtained in each of the regression models. The RR from the univariate adjusted model was 0.22, which implies that the individuals with higher levels of the metabolite have 77% less risk of presenting NAFLD compared to those with lower levels. Similarly, for models 1, 2, 3, and 4, the calculated RR were 0.28, 0.17, 0.25, and 0.28, respectively, which denotes a risk reduction for NAFLD varying from 71 to 82% in individuals with higher levels of ISO compared to subjects with low levels.

## Discussion

NAFLD is a metabolic pathology characterized by the accumulation of fat in the liver parenchyma with an alarmingly growing prevalence [78]. One of the main problems this disease presents is the increased risk of developing potentially deadly complications [35]. Liver biopsy, its diagnostic gold standard, is expensive and invasive, and therefore not suitable for routinary checkups [8]. In this regard, this study yearned to find functional markers that could work as diagnostic tools for NAFLD by using omics technologies. Among the main findings, ISO appeared as a metabolite with such marker characteristics.

To reach this objective, 143 human subjects, either controls or obese subjects with confirmed NAFLD, were enrolled. Metabolic and hepatic status was evaluated and as expected, almost all markers differed between the two groups. Concretely, the hepatic status was significantly worse in the NAFLD group compared to the controls, but these were expected results, since the NAFLD subjects presented hepatic steatosis, while the controls were specifically selected because of their lack of hepatic steatosis. Both diagnoses were confirmed by ultrasonography.

The NAFLD group had overall worst metabolic health compared to controls as well. Surprisingly, the control group showed higher LDL-c and total cholesterol levels despite showing overall better lifestyle and dietary habits; however, similar results were found in previously published literature [43]. Moreover, while the total cholesterol and LDL-c are higher in the control subjects, the LDL-c levels do not reach pathological levels according to both European and American guidelines [22, 42]. Furthermore, HDL-c levels are also higher in control individuals, which could be the reason why total cholesterol is also increased in this group. Low HDL-c levels are highly involved in the development of cardiometabolic diseases and have recently been associated with the development of NAFLD [12, 33, 65]. It is also important to highlight that triglycerides were increased in the NAFLD group. Plasmatic triglycerides are highly involved in the pathophysiology of NAFLD, being responsible for the fatty acid deposition in the hepatocytes which is one of the main causes of the development of the disease [57, 80]. Additionally, both calculated atherogenic indexes were significantly higher in the NAFLD group; suggesting that this group presented not only higher cardiovascular risk compared to the controls but also worst lipidic profiles [16, 39].

HOMA-IR, insulin, and glucose were significantly higher in NAFLD subjects, highlighting the link between the disease and IR, as previously described [70]. Additionally, LECT2 and RBP4 were also increased in these subjects. Similar results to these have been found, which is conceivable since both markers are involved in glucose metabolism, IR, obesity, and metabolic syndrome [31, 32, 54]. LECT2 is a hepatokine that induces the activation of inflammatory cytokines and prevents insulin signaling. Other studies have found that LECT2 is increased in individuals with NAFLD as well and could be considered as a fibrosis marker [24, 32]. RBP4 is associated to cardiovascular risk, inflammation, and insulin resistance, all of which are involved in NAFLD. Other studies have found similar results and suggest that RBP4 could be considered as a biomarker for obesity, metabolic syndrome, and type 2 diabetes [31, 54].

Similarly, leptin levels were also significantly higher in the NAFLD subjects, which has also been found in previous studies. Although leptin produces a satiety signal, it is known that obese individuals have higher concentrations of plasmatic leptin and may have a leptin resistance [26, 55]. In NAFLD concretely, higher leptin levels are associated with greater triglyceride content in the hepatocytes, inflammation, and insulin resistance [5, 45]. Contrarily, adiponectin’s concentration was higher in controls than NAFLD subjects, which was a probable result as well. Adiponectin has been found to be decreased in NAFLD in previous studies too, which has a plausible explanation, since this adipokine is involved in energy metabolism through the reduction of plasmatic triglycerides, increased β-oxidation, and amelioration of glucose metabolism [5, 45].

Dietary characteristics were also quite different among the NAFLD and control groups. Concretely, the NAFLD subjects significantly consumed more calories, carbohydrates, and sodium per day. The glycemic index of the diet was also increased in the NAFLD group compared to the controls. This is relevant since all these variables are associated with the development of insulin resistance and obesity [49, 50, 66]. It is also important to highlight that excessive sodium consumption also leads to other cardiovascular comorbidities, such as high blood pressure, and appears to be a risk factor for mortality, inflammation, and fibrosis in NAFLD patients [20, 56, 80].

Conversely, controls appeared to have higher protein and fat consumption. Slight hyper-proteic diets seem to have a protective effect against the development of obesity, mainly because of its satiating effects and its prevention of muscle loss [51, 71]. On the other hand, higher fat consumption is positively associated with increased body weight and cardiovascular disease [49, 50, 66]. However, the total fat intake in controls is significantly increased in monounsaturated fatty acids (MUFAs) and polyunsaturated fatty acids as well (PUFAs), which are mainly considered beneficial for overall cardiovascular health [14, 67]. Since metabolites are usually a product of diet-derived compounds, the fact that controls possess these significant differences in dietary characteristics compared to NAFLD gives an insight of how and why the serum metabolites differ among the two groups as well [9, 41, 53].

As described in the results, among the discriminant metabolites found between the NAFLD and control groups, four of them were found to be considerably better at predicting the disease through bootstrap stepwise regressions. However, after adjusting for other contributing factors, such as body composition, biochemical, and inflammatory parameters, ISO was considered the main predictive metabolite.

ISO is a bioactive compound found in foods from the Leguminosae/Fabaceae family. It has a polyphenolic structure and is classified as a flavonoid, and more specifically, a chalcone compound [60, 81]. Previous preclinical studies, performed mainly in cellular and animal models, have described its anti-inflammatory, antioxidative, cardiovascular, and liver-protective properties when orally administered [60, 81]. Taking into consideration these previous studies, this metabolite continued to be explored and further analyzed in this sample, since delving into the role of ISO as a biomarker for NAFLD could be of interest given the attributed properties mentioned above.

Furthermore, ISO, the main studied metabolite, correlated negatively not only with hepatic fat but also with key body composition measurements and biochemical parameters involved in NAFLD, specifically those associated with insulin resistance, such as insulin and HOMA-IR.

The observed differences in metabolic health between tertiles could be attributed to lifestyle differences. In fact, although it is not exactly clear why ISO appears to be increased in controls; when examining the lifestyle habits of this group compared to the NAFLD subjects, they do have significantly better dietary habits and increased adherence to the Mediterranean diet, which is characterized by a high consumption of vegetable-based foods, which are rich in these types of bioactive compounds, such as ISO.

Among the food items that contain isoliquiritigenin are garbanzo beans (otherwise known as chickpeas), soybeans, peanuts, and other legumes [52, 60, 81]. It is also found in licorice roots, which are used to make extracts in Chinese medicine [38, 82]. It is worth mentioning that the concentration of ISO may vary among species, and most importantly, there is a need for more research regarding the presence, concentration, and bioavailability of this compound in different food items. In this sense, ISO could also be considered as a biomarker, not only for the following of dietary strategies like the Mediterranean diet but also for the consumption of other produce/vegetables. Additionally, its inclusion could be used for patient feedback to reinforce or encourage healthy dietary behaviors.

Moreover, when divided into tertiles according to ISO intensity, individuals with higher levels presented significantly better body composition and biochemical profiles compared to those with lower levels. These results suggest that this metabolite might act as a protective factor for NAFLD and is overall associated with positive metabolic outcomes. Similar results have been found in previous studies as well [77].

Additionally, the tertile-divided population provided similar information regarding the lifestyle differences of the subjects, since the subjects with higher levels of the metabolite also show significantly better adherence to the Mediterranean diet as well. These results may suggest that a strategy for reaching higher levels of ISO could be sustained by implementing healthy dietary habits. These results are in accordance with previous studies as well, since ISO can be found in foods from the Leguminosae/Fabaceae family, and it seems that it is a diet-derived metabolite that could be considered as a marker for the intake of certain foods [60].

Finally, ISO’s predictive ability was proved with logistic regressions, where the metabolite was significantly evidenced as a predictive protector for NAFLD, even when adjusted and controlled by very potent variables involved in NAFLD as well, such as VAT, adiponectin, plasmatic glucose, and M30. When estimating RR from the OR obtained for easier comprehension [47], individuals with lower levels of the metabolite appeared to have from 71 to 82% of presenting NAFLD compared to those with higher levels.

As mentioned above, ISO is a previously described metabolite that has been studied in cancer, neurological, and liver diseases, including NAFLD, as an administered adjuvant treatment, but not as a biomarker [37, 75, 81]. This molecule is classified as a chalcone, and it is the precursor of several other flavonoids. Chalcones are polyphenols and ISO particularly has two phenyl groups in its structure [83]. Polyphenols are well-known antioxidant agents, capable of scavenging free radicals. Since one of the main mechanisms implied in the pathogenesis of NAFLD is oxidative stress and ISO is a compound with antioxidant properties, the association found in this study has a plausible explanation and has been previously described [40, 69].

Another mechanism in which ISO may exert its antioxidant capacity is by potentiating the nuclear factor, erythroid 2-like 2a (Nrf2) mitochondrial pathway. Nrf2 is a transcription factor critically implicated in the regulation of oxidative stress, inflammation, and autophagy. When attached to the nucleus DNA, Nrf2 seems to enhance the expression of antioxidative genes and their proteins, increasing the production of heme-oxygenase and superoxide dismutase [68, 69].

Recent studies have linked ISO as a protective factor against hepatic steatosis and NAFLD for its ability to increase β-oxidation and inhibit lipogenesis in the liver [72, 81], alleviate insulin resistance, and suppress inflammation [25], mostly through activating protein kinase (AMPK) and peroxisome proliferator-activated receptor alpha (PPAR-α) [75, 77]. PPAR-α is a transcription factor, considered as a major regulator of lipid metabolism in the liver and has a key role in metabolic diseases because of its triglyceride-lowering and insulin-sensitizing properties [4, 73]. AMPK is an enzymatic complex with numerous functions that mainly focus on the regulation of energy metabolism and energy expenditure; it has been proposed as a main mechanism in the pathogenesis of metabolic diseases, such as type 2 diabetes and NAFLD [1, 21].

It is worth noticing that most of the evidence on the effects of ISO are based on studies performed on cellular or animal models. More research, specifically from clinical trials in humans, is needed to further elucidate the role of ISO and its potential role as a biomarker or predictive tool in NAFLD.

It is important to consider that this study has some limitations, such as the lack of mechanistic information about the effect and metabolism of ISO in human physiology. The studies evaluating the role of ISO in humans are scarce, so more information is needed to establish a clear conclusion of the protective role found in this sample.

Nevertheless, this study presents many strengths as well, such as the metabolomic characterization of a sample of human subjects that was extensively characterized with information concerning body composition (measured by DXA), biochemical parameters, lifestyle and dietary habits, and different measures of hepatic variables. Noticeably, all these data were obtained for the controls as well, and in fact, controls were specifically selected because of their confirmed lack of hepatic steatosis. Additionally, the results were verified using a commercial standard of ISO and analyzed using the same conditions as the serum samples.

Furthermore, this study particularly evaluated obese subjects with NAFLD and lean controls, since NAFLD is intrinsically related with obesity and metabolic syndrome, and regressions were adjusted for weight-related confounding variables. However, it could be interesting to evaluate obese subjects without NAFLD as the control group as well and examine ISO in this type of sample. Lastly, it is worth mentioning that this study evaluated the baseline measurements of a particular population sample, but it could be interesting to study the longitudinal changes and behavior of ISO. Since the FLiO study also includes a 24-month dietary intervention of the NAFLD, evaluating the behavior of this metabolite (and possibly others) throughout time via targeted metabolomics could be of interest to continue to elucidate its role on NAFLD.

## Conclusion

This study concludes that metabolites, such as ISO, in combination with visceral adipose tissue, insulin resistance, and other related markers, constitute a potential non-invasive tool to predict and diagnose NAFLD. Individuals with lower levels of ISO have from 71 to 82% more risk of presenting NAFLD compared to individuals with higher levels; therefore, ISO could be considered as a protective predictive factor for NAFLD.

## Supplementary Information

ESM 1 (52.9 KB)
