# Supplementary material for: Isoliquiritigenin in combination with visceral adipose tissue and related markers as a predictive tool for nonalcoholic fatty liver disease
Source: J Physiol Biochem. 2023 Nov 24;80(3):639–53. doi: 10.1007/s13105-023-00998-6 (PMC11502611; doi:10.1007/s13105-023-00998-6)
Supplement: Supplementary file 1 — (52.9 KB) [file 13105_2023_998_MOESM1_ESM.docx]

**Isoliquiritigenin in combination with Visceral Adipose Tissue and related markers as a predictive tool for Nonalcoholic Fatty Liver Disease**

**Short title: Isoliquiritigenin for Nonalcoholic Fatty Liver Disease Management**

Paola Mogna-Peláez^1^. Ana Romo-Hualde^1^. José I. Riezu-Boj^1.2^. Fermin I. Milagro^1.2.3^. David Muñoz-Prieto^1^. José I. Herrero^2.5.6^. Mariana Elorz^2.4^. Alberto Benito-Boillos^2.4^. J. Ignacio Monreal^7^. Josep A. Tur. J^3.8^. Alfredo Martínez^1^. Itziar Abete^1.2.3†^. M. Angeles Zulet^1.2.3†^*.

^1^Department of Nutrition. Food Sciences and Physiology and Centre for Nutrition Research. Faculty of Pharmacy and Nutrition. Centre for Nutrition Research. University of Navarra. 31008 Pamplona. Spain.

^2^Navarra Institute for Health Research (IdiSNA). 31008 Pamplona. Spain.

^3^Biomedical Research Centre Network in Physiopathology of Obesity and Nutrition (CIBERobn). Instituto de Salud Carlos III. 28029 Madrid. Spain.

^4^Department of Radiology. Clínica Universidad de Navarra. 31008 Pamplona. Spain.

^5^Liver Unit. Clínica Universidad de Navarra. 31008 Pamplona. Spain.

^6^Biomedical Research Centre Network in Hepatic and Digestive Diseases (CIBERehd). 28029 Madrid. Spain.

^7^Clinical Chemistry Department. Clínica Universidad de Navarra. 31008 Pamplona. Spain.

^8^Research Group on Community Nutrition and Oxidative Stress. University of Balearic Islands. 07122 Palma. Spain.

^†^These authors contributed equally to this work.

*Authors to whom correspondence should be addressed.

[mazulet@unav.es](mailto:mazulet@unav.es)

ORCID: https://orcid.org/0000-0002-3926-0892

**Supplementary Table 1.** Discriminant putative metabolites obtained through PLSDA analysis.

| **ID** | **Polarity** | **Detected mass**  **(m/z)** | **RT (min)** | **VIP**  **score** | **p-value** | **Intensity (mean)** | | | **Intensity (SD)** | | **Putative metabolite** | **Assignation** | **Δ (mDa)** |
| --- | --- | --- | --- | --- | --- | --- | --- | --- | --- | --- | --- | --- | --- |
|  |  |  |  |  |  | **Control group** | **NAFLD**  **group** | | **Control group** | **NAFLD**  **group** |  |  |  |
| 1 | POS | 460.7414 | 17.06 | 3.81 | 3.83E-16 | 7.98E+03 | | 5.82E+02 | 11497 | 2290 | No found |  |  |
| 2 | POS | 505.6606 | 10.69 | 3.25 | 2.65E-11 | 1.42E+03 | | 5.23E+03 | 2739 | 4141 | No found |  |  |
| 3 | POS | 380.7165 | 16.44 | 3.09 | 4.57E-12 | 3.29E+03 | | 4.61E+02 | 2921 | 1123 | No found |  |  |
| 4 | POS | 372.8361 | 16.98 | 2.75 | 1.26E-10 | 3.10E+03 | | 7.04E+02 | 2517 | 1443 | No found |  |  |
| 5 | POS | 478.1262 | 14.50 | 2.69 | 8.05E-11 | 2.68E+03 | | 8.14E+03 | 2786 | 4858 | 8-Methylthiooctyl glucosinolate | [M+H]^+^ | 2.83 |
|  |  |  |  |  |  |  | |  |  |  | Tetrapepetide Cys Cys Cys Lys | [M+Na]^+^ | 3.93 |
| 6 | POS | 484.1407 | 14.48 | 2.59 | 5.08E-11 | 4.44E+03 | | 1.31E+04 | 4391 | 7734 | 4-(2-Nitroethyl)phenyl primeveroside | [M+Na]^+^ | -1.83 |
| 7 | POS | 372.5007 | 16.98 | 2.43 | 2.54E-10 | 5.88E+03 | | 1.69E+03 | 4257 | 2389 | No found |  |  |
| 8 | POS | 552.5466 | 17.18 | 2.39 | 6.40E-09 | 5.96E+03 | | 1.57E+03 | 5031 | 2338 | No found |  |  |
| 9 | POS | 453.7493 | 16.81 | 2.36 | 1.22E-16 | 2.64E+05 | | 8.20E+04 | 137763 | 54362 | No found |  |  |
| 10 | POS | 463.8687 | 16.99 | 2.36 | 8.99E-12 | 9.84E+03 | | 3.09E+03 | 6179 | 4069 | No found |  |  |
| 11 | POS | 650.3224 | 16.98 | 2.35 | 1.81E-19 | 6.43E+04 | | 2.21E+04 | 25381 | 11459 | Tetrapeptide Leu Pro Glu Phe Tyr | [M+H-H_2_O]^+^ | 3.41 |
| 12 | POS | 282.2828 | 18.45 | 2.35 | 7.17E-19 | 3.08E+04 | | 9.35E+04 | 14420 | 49555 | L-threo-Sphingosine C-18 | [M+H-H_2_O]^+^ | 3.07 |
|  |  |  |  |  |  |  | |  |  |  | Oleamide | [M+H]^+^ | 3.67 |
| 13 | POS | 453.2484 | 16.81 | 2.32 | 1.41E-16 | 5.44E+05 | | 1.74E+05 | 284787 | 109114 | Rhodojaponin I | [M+H]^+^ | 0.07 |
| 14 | POS | 454.7539 | 16.81 | 2.31 | 4.87E-17 | 1.92E+04 | | 6.22E+03 | 9568 | 4013 | No found |  |  |
| 15 | POS | 536.2748 | 17.04 | 2.26 | 9.41E-18 | 1.14E+05 | | 4.13E+04 | 43246 | 21497 | Vignatic acid A | [M+H-H_2_O]^+^ | -1.30 |
| 16 | POS | 440.1754 | 14.48 | 2.26 | 1.38E-12 | 9.76E+04 | | 3.04E+05 | 80672 | 184028 | Tetrapeptide Asp Leu Asn Gly | [M+Na]^+^ | 0.18 |
| 17 | POS | 780.2767 | 16.80 | 2.25 | 1.92E-11 | 2.17E+03 | | 6.44E+03 | 2224 | 3922 | 3'-Sialyl-3-fucosyllactose | [M+H]^+^ | -0.08 |
| 18 | POS | 558.7481 | 16.98 | 2.25 | 1.50E-11 | 7.48E+03 | | 2.54E+03 | 4550 | 2651 | No found |  |  |
| 19 | POS | 502.8803 | 17.10 | 2.23 | 3.41E-07 | 6.43E+03 | | 2.22E+03 | 5798 | 4312 | 6.14.16-Tribromo-5Z.7E.9E.13E.15Z-hexadecapentaenoic acid | [M+Na]^+^ | -2.41 |
| 20 | POS | 456.1465 | 14.49 | 2.21 | 1.75E-14 | 2.44E+04 | | 6.98E+04 | 14289 | 35338 | Tripeptide Asp Tyr His | [M+Na]^+^ | -2.48 |
| 21 | POS | 434.8581 | 17.00 | 2.21 | 6.75E-08 | 3.05E+03 | | 9.74E+02 | 2502 | 1794 | 3-(3.5-Diiodo-4-hydroxyphenyl)lactate | [M+H]^+^ | -0.41 |
| 22 | POS | 300.2937 | 18.45 | 2.16 | 5.00E-16 | 9.45E+04 | | 2.63E+05 | 53300 | 162842 | Phytosphingosine | [M+H-H_2_O]^+^ | 3.41 |
|  |  |  |  |  |  |  | |  |  |  | 2-Amino-octadecanoic acid | [M+H]^+^ | 4.01 |
| 23 | POS | 1226.9843 | 21.75 | 2.15 | 1.46E-06 | 7.62E+05 | | 2.47E+05 | 717354 | 298625 | No found |  |  |
| 24 | POS | 709.9339 | 16.77 | 2.15 | 1.73E-07 | 3.37E+03 | | 9.21E+03 | 5695 | 8248 | No found |  |  |
| 25 | POS | 624.7764 | 16.99 | 2.13 | 7.48E-10 | 4.18E+03 | | 1.55E+03 | 2331 | 1674 | No found |  |  |
| 26 | POS | 530.7181 | 17.20 | 2.13 | 3.81E-12 | 5.53E+03 | | 1.93E+03 | 3387 | 1570 | No found |  |  |
| 27 | POS | 442.1771 | 14.48 | 2.12 | 4.03E-11 | 4.88E+03 | | 1.31E+04 | 3687 | 7884 | Tetrapeptide Ala Ala Met Gln | [M+Na]^+^ | 4.00 |
| 28 | POS | 379.7126 | 16.35 | 2.09 | 2.13E-15 | 4.69E+04 | | 1.75E+04 | 22306 | 12495 | No found |  |  |
| 29 | POS | 265.2591 | 24.23 | 2.08 | 7.52E-06 | 4.15E+04 | | 1.24E+05 | 68414 | 102460 | No found |  |  |
| 30 | POS | 434.5237 | 17.00 | 2.07 | 2.96E-08 | 4.81E+03 | | 1.79E+03 | 3524 | 2682 | No found |  |  |
| 31 | POS | 408.1548 | 10.42 | 2.05 | 1.47E-07 | 2.23E+04 | | 7.32E+03 | 18697 | 9610 | Tetrapeptide Ser Ala Cys Gln | [M+H]^+^ | 0.13 |
| 32 | POS | 463.5336 | 16.99 | 2.03 | 4.36E-10 | 1.37E+04 | | 4.69E+03 | 8880 | 5539 | No found |  |  |
| 33 | POS | 530.7427 | 17.06 | 2.02 | 2.21E-12 | 7.66E+03 | | 2.90E+03 | 4070 | 2221 | No found |  |  |
| 34 | POS | 694.7991 | 17.00 | 1.97 | 7.87E-11 | 2.13E+04 | | 7.66E+03 | 12945 | 7880 | No found |  |  |
| 35 | POS | 415.5170 | 16.97 | 1.97 | 1.75E-10 | 4.48E+04 | | 1.71E+04 | 26310 | 16949 | No found |  |  |
| 36 | POS | 415.8502 | 16.97 | 1.96 | 4.43E-10 | 2.80E+04 | | 1.07E+04 | 16804 | 10792 | No found |  |  |
| 37 | POS | 1228.9926 | 21.75 | 1.95 | 2.35E-06 | 2.20E+05 | | 7.23E+04 | 200406 | 84834 | No found |  |  |
| 38 | POS | 457.1490 | 14.47 | 1.93 | 6.81E-11 | 4.90E+03 | | 1.28E+04 | 3034 | 7563 | Steganacin | [M+H]^+^ | -0.32 |
|  |  |  |  |  |  |  | |  |  |  | Luteolin 7.3'-dimethyl ether 5-glucoside | [M+H-H_2_O]^+^ | -0.92 |
|  |  |  |  |  |  |  | |  |  |  | Tetrapeptide Asn Gln Cys Ala | [M+Na]^+^ | 1.38 |
|  |  |  |  |  |  |  | |  |  |  | Tetrapeptide Cys Asp His Thr | [M+H-H_2_O]^+^ | -1.52 |
|  |  |  |  |  |  |  | |  |  |  | Vestitone 7-glucoside | [M+Na]^+^ | 2.08 |
| 39 | POS | 343.2691 | 24.41 | 1.80 | 4.23E-04 | 8.93E+05 | | 3.14E+05 | 851592 | 499409 | No found |  |  |
| 40 | POS | 572.9179 | 16.88 | 1.69 | 1.37E-08 | 6.79E+04 | | 2.60E+04 | 76046 | 23838 | No found |  |  |
| 41 | POS | 291.0719 | 10.41 | 1.67 | 9.42E-07 | 7.73E+04 | | 2.94E+04 | 60806 | 26725 | N.N-Didemethylchlorpromazine | [M+H]^+^ | 0.21 |
|  |  |  |  |  |  |  | |  |  |  | Inosine | [M+Na]^+^ | 1.91 |
| 42 | POS | 880.4595 | 16.89 | 1.64 | 4.81E-07 | 5.37E+04 | | 2.01E+04 | 59450 | 21733 | No found |  |  |
| 43 | POS | 317.2570 | 23.51 | 1.63 | 1.30E-03 | 5.61E+05 | | 2.15E+05 | 926897 | 422311 | No found |  |  |
| 44 | POS | 1256.9696 | 20.99 | 1.61 | 1.26E-04 | 7.26E+05 | | 2.73E+05 | 1022000 | 491822 | No found |  |  |
| 45 | POS | 1257.9697 | 20.99 | 1.59 | 1.49E-05 | 3.27E+05 | | 1.24E+05 | 412113 | 206748 | No found |  |  |
| 46 | POS | 810.5929 | 20.03 | 1.36 | 1.65E-03 | 2.10E+06 | | 7.09E+06 | 3527200 | 8673513 | No found |  |  |
| 47 | POS | 699.9560 | 20.13 | 1.19 | 7.77E-05 | 2.81E+04 | | 7.81E+04 | 14577 | 89459 | No found |  |  |
| 48 | POS | 412.7679 | 9.12 | 1.04 | 8.06E-05 | 6.83E+03 | | 2.38E+04 | 1664 | 37373 | No found |  |  |
| 49 | NEG | 601.7628 | 17.04 | 2.19 | 3.65E-10 | 2.88E+04 | | 9.24E+03 | 23043 | 14428 | No found |  |  |
| 50 | NEG | 513.1417 | 16.82 | 2.08 | 4.14E-13 | 2.65E+03 | | 1.21E+04 | 1850 | 11889 | 2''.6''-Di-O-acetylononin | [M-H]^-^ | 1.50 |
|  |  |  |  |  |  |  | |  |  |  | 5.7-Dihydroxy-6-C-methylflavone 7-xylosyl-(1->3)-xyloside | [M-H_2_O-H]^-^ | 2.00 |
| 51 | NEG | 534.2467 | 17.04 | 1.98 | 1.72E-12 | 3.18E+04 | | 1.05E+04 | 20982 | 6738 | Tripeptide Trp His Pro Pro | [M-H]^-^ | -0.29 |
| 52 | NEG | 486.1355 | 17.06 | 1.98 | 3.75E-10 | 6.01E+03 | | 3.00E+04 | 3913 | 32256 | No found |  |  |
| 53 | NEG | 319.2111 | 20.13 | 1.87 | 2.89E-09 | 1.04E+04 | | 3.86E+04 | 10453 | 36206 | 11.17-Dihydroxy-9-fluoro-17-methyl-5-androstan-3-one | [M-H_2_O-H]^-^ | 3.84 |
|  |  |  |  |  |  |  | |  |  |  | Santalyl phenylacetate | [M-H_2_O-H]^-^ | 4.94 |
| 54 | NEG | 534.9220 | 20.27 | 1.59 | 6.70E-08 | 2.91E+03 | | 7.99E+03 | 5504 | 13897 | No found |  |  |
| 55 | NEG | 255.0695 | 17.83 | 1.39 | 3.23E-06 | 2.40E+05 | | 8.29E+04 | 308809 | 49615 | Isoliquiritigenin | [M-H]^-^ | 3.18 |
| 56 | NEG | 211.0823 | 17.84 | 1.36 | 9.44E-06 | 1.37E+05 | | 4.80E+04 | 180123 | 27362 | Perseitol | [M-H]^-^ | -0.02 |
| 57 | NEG | 418.0534 | 23.96 | 1.07 | 1.88E-03 | 5.38E+03 | | 1.87E+04 | 6567 | 49322 | No found |  |  |

Abbreviations: m/z, mass charge ratio; RT (min), Retention Time (minutes); VIP, Variable Importance in Projection; Δ (mDa), Variation of mDa between theoretical and detected mass. Data represents mean and standard deviation (SD) of the intensity of different metabolites found through non-targeted metabolomics. P-values and VIP were obtained through PLSDA analysis. Assignation refers to the adducts used to identify the metabolite in the Metlin database.
